# Evidence That Obesity Risk Factor Potencies Are Weight Dependent, a Phenomenon That May Explain Accelerated Weight Gain in Western Societies

**DOI:** 10.1371/journal.pone.0027657

**Published:** 2011-11-23

**Authors:** Paul T. Williams

**Affiliations:** Lawrence Berkeley National Laboratory, Berkeley, California, United States of America; Institut Pluridisciplinaire Hubert Curien, France

## Abstract

**Background:**

We have shown that individuals at the highest percentiles of the body mass index (BMI) distribution (i.e., most overweight) experience greater increases in body weight from sedentary lifestyle than those from the lowest percentiles. The purpose of the current analyses was to assess whether recent, accelerated increases in obesity could potentially be due to increased vulnerability to obesity risk factors as the population has become more overweight.

**Methodology/Principal Findings:**

Quantile regression was used to compare BMI population percentiles to obesity risk factors (lower education, diets characterized by high-meat/low-fruit content, parental adiposity) in two independent samples of men (N_1_ = 3,513, N_2_ = 11,365) and women (N_1_ = 15,809, N_2_ = 10,159). The samples were subsets of the National Walkers' (Study 1) and Runners' (Study 2) Health Studies whose physical activities fell short of nationally recommended activity levels. The data were adjusted for age, race, and any residual effects of physical activity. The regression slopes for BMI vs. education, diet, and family history became progressively stronger from the lowest (e.g., 5^th^, 6^th^…) to the highest (e.g., …, 94^th^, 95^th^) BMI percentiles. Compared to the 10^th^ BMI percentile, their effects on the 90^th^ BMI percentile were: 1) 2.7- to 8.6-fold greater in women and 2.0- to 2.4-fold greater in men for education; 2) 3.6- to 4.8-fold greater in women and 1.7- to 2.7-fold greater in men for diet; and 3) 2.0- to 2.6-fold greater in women and 1.7-fold greater in men for family history.

**Conclusions/Significance:**

Thus we propose risk factors that produce little weight gain in lean individuals may become more potent with increasing adiposity. This leads us to hypothesize that an individual's obesity is itself a major component of their obesogenic environment, and that, the cycle of weight gain and increased sensitivity to obesity risk factors may partly explain recent increases in obesity in western societies.

## Introduction

Obesity (body mass index, BMI, ≥30 kg/m^2^) has increased from 15% to 33% in U.S. adults between 1980 and the early 2000 s [1], and is projected to affect over 50% by 2030 [2]. The obesity epidemic has been most often ascribed to the confluence of two factors: 1) the emergence of the obesogenic environment, consisting of diminished physical activity and the availability and promotion of inexpensive, palatable, energy-dense foods [3]; and 2) a genetic legacy that favors storing fat in anticipation of times of food scarcity [4], [5]. The epidemic is hypothesized to be due to the mal-adaptation of this genetic legacy to the obesogenic environment [6]. Although other factors may also contribute to the recent rise in obesity [7], [8], there appears to be widespread consensus regarding the importance of the obesogenic environment [9].

We have argued that the percentile distribution of BMI in the population is an important framework for understanding obesity [10]–[14]. The increases in BMI over the last several decades have not been distributed evenly throughout the BMI distribution, but rather have been proportionately greater for the higher BMI population percentiles [9], [15]. The 24% increase in obesity between 2000 and 2005 included a 50% increase in BMI≥40 kg/m^2^ and a 75% increase in BMI≥50 kg/m^2^ [16]. Prospectively, weight gain tends to be greater in overweight (25 kg/m^2^≤BMI<30 kg/m^2^) and obese (BMI ≥30 kg/m^2^) subjects compared with healthy-weight subjects (18 kg/m^2^≤BMI<25 kg/m^2^) [15]. The apparent effects of both moderate-intensity (e.g., walking) and vigorous-intensity (e.g., running) physical activity on BMI are substantially greater at the higher percentiles of the BMI distribution [10]–[14].

A recent series of papers has demonstrated that the increase in BMI associated with sedentary lifestyle is substantially greater among individuals at the higher percentiles of the BMI distribution than among lean individuals [10]–[14]. Other obesity risk factors may also become progressively more potent with increasing adiposity [17]–[23]. This suggests to us that an individual's obesity may itself be or become a significant part of their obesogenic environment, and the epidemic of obesity in the population could be the product of a cycle of weight gain and increased sensitivity to obesity risk factors in its members.

We therefore examined the relationships of obesity risk factors to the percentile distribution of BMI (e.g., 5^th^, 6^th^… 95^th^ percentiles) using quantile regression [24]. The technique was used to provide robust, distribution-free tests of whether the effects of obesity risk factors become progressively stronger with the increasing percentile of the BMI distribution. Together with our previous published results [10]–[14], these analyses provide cross-sectional support of the hypothesis that a broad range of risk factors (low educational attainment, diet, inheritance, sedentary lifestyle) have substantially greater affect on the higher (i.e., more overweight) than the lower (leaner) BMI percentiles.

## Results

Table 1 presents the risk factor distribution for males and females separately in the two samples. Table 2, which displays the characteristics of the samples by BMI, shows that higher BMI was also associated with fewer years of education, greater parental adiposity, and diets characterized by greater meat and lower fruit consumption.

**Table 1:** Distribution of obesity risk factors in two independent samples.

	Females		Males	
	Study 1	Study 2	Study 1	Study 2
N*	15,809	10,159	3,513	11,365
Education (% of sample)				
<12 years	0.5	0.3	0.8	0.2
12–15.9 years	39.2	22.8	25.7	19.9
16–19.9 years	55.5	69.0	56.1	64.1
≥20 years	4.8	7.8	17.4	15.8
Mother's reported adiposity (% of sample)				
Lean	16.1	15.2	18.3	17.0
Average	39.7	40.3	44.8	42.0
Overweight	35.3	35.0	31.1	32.8
Very overweight	8.9	9.5	5.9	8.3
Father's reported adiposity (% of sample)				
Lean	23.9	23.0	21.7	18.5
Average	46.1	43.8	49.2	46.6
Overweight	25.0	27.4	25.6	29.4
Very overweight	5.1	5.8	3.5	5.5
Meat consumption (% of sample)				
0 servings/day	15.0	24.2	11.2	10.8
0.01 to 0.5 servings/day	56.8	56.2	51.4	53.5
0.51–1.0 servings/day	24.4	16.9	30.3	27.9
>1 serving/day	3.9	2.7	7.1	7.8
Fruit consumption (% of sample)				
0 pieces/day	2.8	2.4	3.7	3.4
0.1 to 1.0 pieces/day	39.7	48.1	44.0	50.0
1.1 to 2.0 pieces/day	33.8	32.2	30.7	29.2
2.1 to 3.0 pieces/day	17.6	13.1	15.1	12.6
>3.0 pieces/day	6.1	4.2	6.5	4.7

*Parental adiposities were requested only during initial Study 2 recruitment and are therefore available for 2,721 women and 5,807 men in that study.

**Table 2:** Characteristics of the samples by body mass index.

	Body mass index (BMI), kg/m^2^				
	<22.5	22.5 to 24.9	25 to 27.4	27.5 to 29.9	≥30
*Sample size*					
*Study 1-females*	4040	3638	3019	1720	3392
*Study 2-females*	6023	2388	1020	361	367
*Study 1-males*	323	719	983	650	838
*Study 2-males*	1998	3834	3295	1408	830
Age (years)					
*Study 1-females*	48.79±13.26	51.12±12.83	51.48±12.86	51.46±12.94	49.80±12.19
*Study 2-females*	37.37±10.34	39.36±10.54	40.72±10.55	40.91±10.40	42.18±10.27
*Study 1-males*	60.71±13.72	61.12±12.26	60.22±12.57	60.17±11.40	57.00±11.46
*Study 2-males*	42.34±13.27	44.88±11.77	45.28±10.60	45.17±9.61	45.12±9.92
Education (years)					
*Study 1-females*	15.57±2.47	15.36±2.50	15.18±2.51	14.98±2.56	14.91±2.54
*Study 2-females*	16.12±2.32	16.07±2.33	15.94±2.36	15.77±2.39	15.44±2.61
*Study 1-males*	16.75±2.63	16.60±2.69	16.31±2.74	16.05±2.79	15.84±2.73
*Study 2-males*	16.57±2.58	16.64±2.46	16.43±2.41	16.29±2.46	16.17±2.53
Dietary index*					
*Study 1-females*	0.47±0.73	0.55±0.72	0.65±0.73	0.72±0.83	0.80±0.85
*Study 2-females*	0.20±0.32	0.24±0.36	0.26±0.33	0.36±0.45	0.33±0.42
*Study 1-males*	−0.03±0.72	0.13±0.70	0.21±0.76	0.35±0.78	0.46±0.76
*Study 2-males*	0.06±0.34	0.08±0.32	0.12±0.32	0.15±0.37	0.17±0.34
Parental adiposity index†					
*Study 1-females*	3.18±0.93	3.30±0.93	3.43±0.93	3.49±0.94	3.63±0.98
*Study 2-females*	1.84±0.55	1.98±0.55	2.07±0.60	1.94±0.60	2.11±0.61
*Study 1-males*	2.78±0.81	2.90±0.79	3.05±0.79	3.14±0.83	3.33±0.88
*Study 2-males*	1.92±0.59	2.05±0.63	2.13±0.62	2.21±0.62	2.26±0.60

*adjusted for age, education, and exercise.

† adjusted for age, education, diet, and exercise.

### Educational attainment

The standard regression estimates of the decreases in BMI per year of education (i.e., ignoring the differences between percentiles, slope±SE) were −0.180±0.017 kg/m^2^ for Study 1 and −0.078±0.014 kg/m^2^ for Study 2 females, and −0.193±0.027 kg/m^2^ for Study 1 and −0.096±0.012 kg/m^2^ for Study 2 males when adjusted for age, race, exercise, diet and parental adiposity as described under Methods.

**Figure 1:**
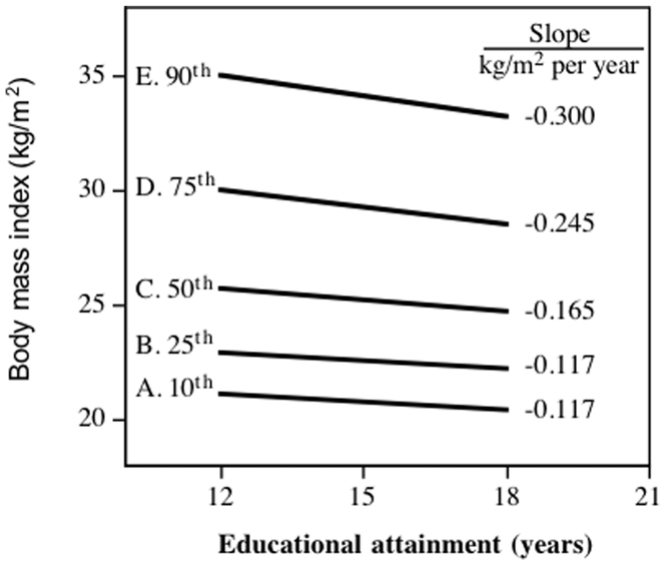
Regression lines comparing the calculated per kg/m^2^ decrease in BMI per year of educational attainment in Study 1 females, where BMI is represented along the Y-axis and reported educational attainment is represented along the X-axis. Data adjusted for age, race, physical activity, parental adiposity, and diet. Individual slopes significantly different from zero at p<10^−6^.

**Figure 2:**
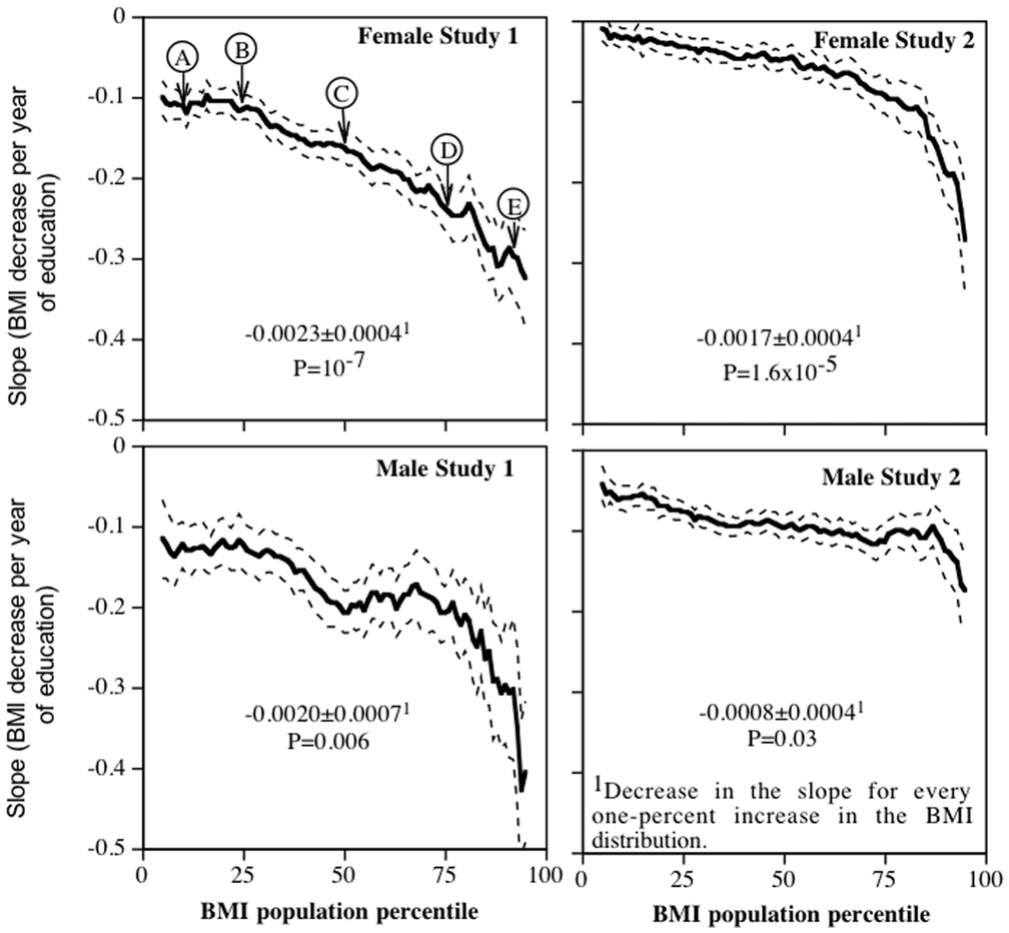
Percentile plot showing the slope for BMI vs. years of education (Y-axis) at each percentile of the BMI distribution (X-axis). For example, each addition year of education in Study 1 females was associated with a BMI decrease of (slope±SE) −0.117±0.018 kg/m^2^ at the 10^th^ percentile of their BMI distribution (A), −0.117±0.016 kg/m^2^ at their 25^th^ percentile (B), −0.165±0.020 kg/m^2^ at their 50^th^ percentile (C, the median), −0.245±0.029 kg/m^2^ at their 75^th^ percentile (D), and −0.300±0.046 kg/m^2^ at their 90^th^ percentile (E, compare with Figure 1). The dashed lines designate one standard error. Data adjusted for age, race, physical activity, and diet. Study 1 included additional adjustment for parental adiposity.

Figure 1 presents the decline in BMI per year of education in Study 1 women at the 10^th^ 25^th^, 50^th^, 75^th^ and 90^th^ percentile of the BMI distribution (all P<10^−10^). These slopes, along with the slopes for the other BMI percentiles from the quantile regression analyses, were used to create Figure 2. The Y-axis of Figure 2 represents the apparent effect (i.e. slope) of each 1-year increase in education on the 5^th^ percentile of the BMI distribution, 6^th^ percentile of the BMI distribution,…, and the 95^th^ percentiles of the BMI distribution, where the percentiles are plotted along the X-axis. Dashed lines present the corresponding standard errors at each percentile. The Y-axis is the slope of the decrease in BMI per year of education, rather than BMI itself (compare with Figure 1). If the slope relating BMI to education was the same throughout the BMI distribution, as assumed by most statistical tests, then the slopes of the lines in Figure 1 would be parallel, and the plot in Figure 2 would be a simple horizontal line. In fact, Figure 2 shows that the increase in BMI became progressively stronger with increasing percentiles of the BMI distribution, such that on average each 1-percent increase in the BMI distribution was associated with a 0.0023±0.0004 kg/m^2^ greater reduction in BMI per year of education. The BMI reduction per year of education was 2.67-fold greater at the 90^th^ BMI percentile that the 10^th^ BMI percentile. The difference in slope between the 10^th^ and 90^th^ percentile (−0.184 kg/m^2^ per year) was as large as the traditional standard regression estimate of a 0.180 kg/m^2^ decrease in BMI per year of education for the entire sample. The graphs also demonstrate the inadequacy of the standard regression analyses to estimate the decline in BMI per year of education, i.e., the 95^th^ confidence interval for the standard regression slope (i.e., ±1.96*SE) includes only those slopes between the 37^th^ and 67^th^ percentiles of the BMI distribution. In other words, standard regression estimates misrepresent the effect of education on female BMI for 69% of the Study 1 sample.

Figure 2 also presents the corresponding analyses of education in Study 2 women and Study 1 and 2 men. Again, the Y-axis refers to the calculated effect (i.e., slope) of a 1-year increase in education on BMI, not BMI itself. On average, each one percent increase in the BMI percentile was associated with a 0.0017±0.0004 kg/m^2^ greater decrease in the slope in Study 2 females (P<0.0001), and 0.0020±0.0007 kg/m^2^ and 0.0008±0.0004 kg/m^2^ greater decreases in the slopes in Study 1 and 2 males, respectively. Compared to the slope for BMI vs. education at the 10^th^ BMI percentile, the slope at the 90^th^ BMI percentile was 8.65-fold greater for Study 2 females, and 2.42- and 2.03-fold greater for Study 1 and 2 males, respectively. The greater reduction in BMI per education year in Study 1 than Study 2 women is consistent with: 1) the overall greater BMI in Study 1 than Study 2 women, and 2) the progressively greater effect of education in heavier vs. leaner women as shown in both graphs. The 95^th^ confidence interval for the standard regression slopes cited above includes only the 54 to 79 percentiles of the BMI distribution for Study 2 women (i.e., misrepresenting 74% of the sample) and include only the 35 to 82 and 22 to 71 percentiles of the BMI distribution for Study 1 and 2 men, respectively (i.e., misrepresenting 52% and 50% of the sample, respectively).

### Diet

The standard regression estimates of the increases in BMI per increase in the dietary index (slope±SE) were 1.000±0.054 kg/m^2^ and 1.000±0.094 kg/m^2^ in Study 1 and Study 2 women, respectively, and 1.000±0.099 kg/m^2^ and 1.000±0.090 kg/m^2^ in Study 1 and Study 2 men, respectively. The slopes are all exactly one because the indices were derived from the regression analyses of these data (see Methods).

**Figure 3:**
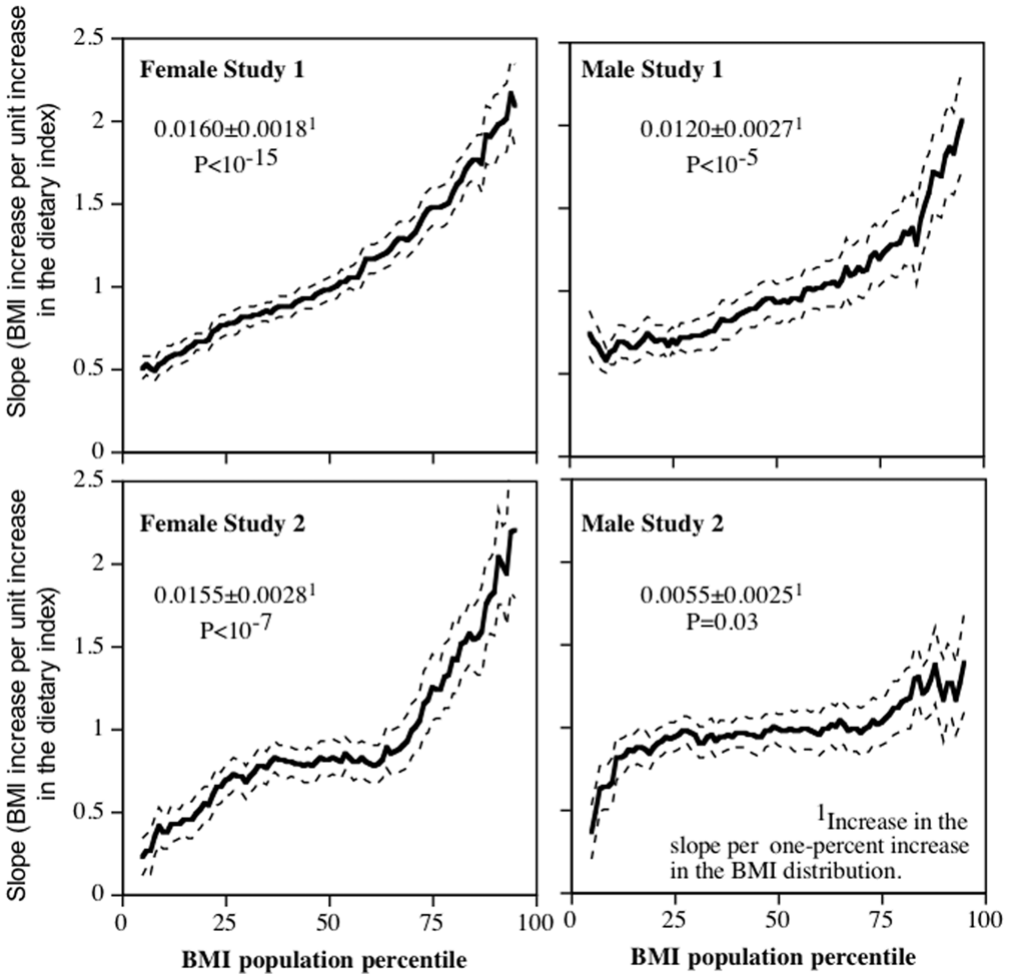
Percentile plot showing the slope for BMI vs. the dietary index (high-meat/low-fruit content, Y-axis) at each percentile of the BMI distribution (X-axis). The exact definitions of the dietary indices are described in the Methods Section. The dashed lines designate one standard error. Data adjusted for age, race, and education. Study 1 included additional adjustment for parental adiposity.

Figure 3 shows that the magnitude of the BMI increase per unit increase in the diet index increased progressively with increasing percentiles of the BMI distribution. On average, each one percent increase in the BMI percentile was associated with a 0.0160±0.0018 kg/m^2^ and 0.0155±0.0028 kg/m^2^ greater increases in the slope in Study 1 and 2 women, respectively, and 0.0120±0.0027 kg/m^2^ and 0.0055±0.0025 kg/m^2^ greater increases in the slope in Study 1 and 2 men, respectively. Compared to the slope for BMI vs. diet index at the 10^th^ BMI percentile, the slope at the 90^th^ BMI percentile was 3.60-fold and 4.78-fold greater in Study 1 and 2 women, respectively, and 2.73-fold and 1.74-fold greater in Study 1 and 2 men, respectively. The 95^th^ confidence interval for the standard regression slopes cited above includes only the 43^th^ to 58^th^ and the 37^th^ to 74^th^ percentiles of the BMI distribution for Study 1 and 2 women, respectively (i.e., misrepresenting 84% and 62% of their respective samples) and include only the 37^th^ to 72^th^ and 13^th^ to 81^st^ percentiles of the BMI distribution for Study 1 and 2 men, respectively (i.e., misrepresenting 64% and 31% of their respective samples).

### Family history

The standard regression estimates of the BMI increases per increase in parental adiposities (slope±SE) were 1.000±0.044 kg/m^2^ and 1.000±0.114 kg/m^2^ in Study 1 and Study 2 women, respectively, and 1.000±0.089 kg/m^2^ and 1.000±0.071 kg/m^2^ in Study 1 and Study 2 men, respectively.

**Figure 4:**
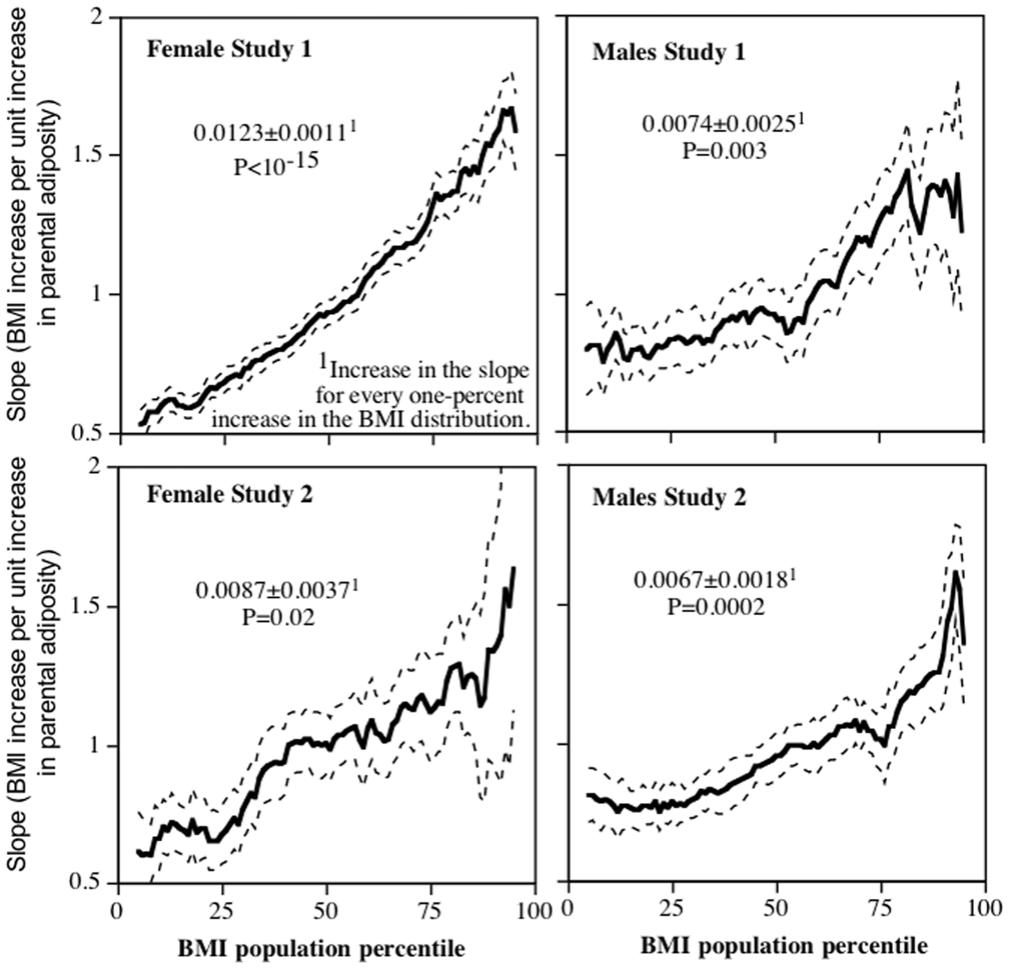
Percentile plot showing the slope for BMI vs. parental adiposity (Y-axis) at each percentile of the BMI distribution (X-axis). The dashed lines designate one standard error. Data adjusted for age, race, education, physical activity, and diet.

Figure 4 shows that the magnitude of the slope for BMI vs. parental adiposity also increased progressively with increasing percentiles of the BMI distribution. On average, each one percent increase in the BMI percentile was associated with 0.0123±0.0011 and 0.0087±0.0037 kg/m^2^ greater increases in the slope in Study 1 and 2 women, respectively, and 0.0074±0.0025 and 0.0067±0.0018 kg/m^2^ greater increases in the slopes in Study 1 and 2 men, respectively. Compared to the slope for BMI vs. parental adiposity at the 10^th^ BMI percentile, the slope at the 90^th^ BMI percentile was 2.65-fold and 2.03-fold greater in Study 1 and 2 women, respectively, and 1.71-fold and 1.67-fold greater in Study 1 and 2 men, respectively. The 95^th^ confidence interval for the standard regression slopes cited above includes only the 48^th^ to 60^th^ and the 31^st^ to 78^th^ percentiles of the BMI distribution for Study 1 and 2 women, respectively (i.e., misrepresenting 87% and 52% of their respective samples) and includes only the 12^th^ to 69^th^ and 42^nd^ to 79^st^ percentiles of the BMI distribution for Study 1 and 2 men, respectively (i.e., misrepresenting 42% and 62% of their respective samples).

## Discussion

These analyses show that the regression slopes for BMI vs. education, diet, and family history became progressively stronger from the lowest (e.g., 5^th^, 6^th^…) to the highest (e.g., …, 94^th^, 95^th^) BMI percentiles. Compared to the 10^th^ BMI percentile, their effects on the 90^th^ BMI percentile were: 1) 2.7- to 8.6-fold greater in women and 2.0- to 2.4-fold greater in men for education; 2) 3.6- to 4.8-fold greater in women and 1.7- to 2.7-fold greater in men for diet; and 3) 2.0- to 2.6-fold greater in women and 1.7-fold greater in men for family history. The trends in the regression slope with increasing percentiles of the BMI distribution were statistically significant for both sexes, and in two separate cohorts.

We have previously demonstrated that the effect of physical inactivity or sedentary lifestyle, a major risk factor for obesity, increases in proportion to the percentile of the BMI distribution, such that the associated weight increase is substantially greater in overweight than lean individuals [10]–[14]. Per kilometer run per week, the associated decline for BMI was three-fold greater in men, and 6-fold greater in women, at the 95th than at the 5th BMI percentile in male and female runners [10]. Additional studies of runners confirmed that the inverse association between physical activity and BMI was proportional to the percentile of the BMI distribution [13], [14]. Among walkers, increasing walking distance from 10 to 11 km/wk was associated with a 15-fold greater decrease in women's BMI at the 95^th^ than the 5^th^ BMI percentiles [12]. In men, the decline in BMI per km/day walked ranged from 4.9- to 6-fold greater at the 90^th^ vis-à-vis the 10^th^ BMI percentile [11].

These earlier analyses, in conjunction with the current results, suggest that the effects of four major obesity risk factors on BMI become progressively greater with increasing percentiles of the BMI distribution: low educational attainment [9], family history of excess body weight [25], [26], diets characterized by high meat and low fruit intake [15], and physical inactivity [27], [28]. The different effects in lean (e.g., 10^th^ BMI percentile) versus overweight individuals (e.g., 90^th^ BMI percentile) were nontrivial, i.e., ranging from 2.0-fold to 8.7-fold greater effect for education, from 1.7-fold to 4.8-fold greater effect for diet, from 1.7-fold to 2.7-fold greater effect for inheritance, and as much as a 15-fold greater effect for physical inactivity. In fact, in every case the difference between the 10^th^ and 90^th^ BMI percentile exceeded the standard regression estimate. Thus, describing these BMI-risk factor relationships in term of their different effects in lean and overweight individuals is as important as characterizing the entire sample by their standard regression estimate.

The results are entirely consistent with our hypothesis that the risks for weight gain due to low socioeconomic status, diet, inheritance and physical inactivity are minor in relatively lean individuals, and become progressively greater with increasing BMI. The compounding effect of the risk factors with ever-increasing obesity will accelerate weight gain, which may explain, in part, the epidemic rise of obesity in the United States and elsewhere. Thus we hypothesized that obesity itself may be a major, if not the most important, attribute defining the obesogenic environment. This leads us to hypothesize that obesity itself may be a key element of an individual's obesogenic environment, which may explain, in part, the epidemic rise of obesity in the United States and elsewhere.

Although the average BMI has increased gradually over the past 100 years in the United States [29], its rise has accelerated sharply since about the mid-1980 s [9]. The acceleration is assumed to correspond to the emergence of an obesogenic environment [3]. Greater calorie consumption has been attributed to aggressive marketing of high-fat, energy-dense foods and large portion sizes served outside the home [9]. In addition, consumption of high-fructose corn syrup increased over 10-fold between 1970 and 1990 [30]. Fructose metabolized by the liver favors de novo lipogenesis, and fructose may signal satiety less effectively than the glucose it replaced [30]. Greater inactivity has been attributed to decreased manual and household labor and more time sitting during leisure, work, and commuting without scheduled regular exercise [31]. It is hypothesized that obesity arises from the imposition of these environmental conditions onto genes evolved to store energy, genes maladapted to the obesogenic environment [6]. The thrifty gene hypothesis postulates that these genes evolved to increase survival during famines [4]. Others hypothesize that humans evolved to more effectively store fructose as body fat by losing the ability to synthesize vitamin C and degrade uric acid [5].

Education and genetic inheritance of parental adiposity are not generally considered factors that have changed recently. However, our theory suggests that their contributions to obesity have intensified due to the greater corpulence of the population and the increasing potency of low educational attainment and inherited adiposity with body weight. The significant linear increase in the potency of four distinct risk factors with adiposity suggests that this phenomenon could apply broadly to other obesity risk factors. Traditionally, the etiology of the obesity epidemic has been evaluated in terms of whether there has been an increase in the prevalence of a risk factor that corresponds temporally to the increase in obesity [7]–[9]. The theory proposed in this paper hypothesizes that the obesity epidemic could be caused by risk factors whose prevalence has remained constant, but whose effects are magnified by the increased corpulence of the population. Diet and inactivity may have instigated the trend towards greater corpulence, which then accelerated due to the amplification of the effects of other factors.

The proposed theory may explain, in part, why only 10% of normal weight children become obese adults while ≥75% of obese children become obese adults [32]–[34]. Specifically, obese children are already sensitized to the risk factors for obesity whereas normal weight children are not. The theory does not account for the rapid increase in body weight in dieters who relapse, whose rapid return to pre-dieting weight would suggest greater susceptibility to obesity risk factors despite reducing overall body fat [9]. Obesity is the result of both increased adipocyte size and number [35]. Over 80% of the patients who intentionally lose weight regain the weight lost [6]. The abruptness of weight gain in these individuals is substantially greater than the gradual weight increase of the population, suggesting that different physiological mechanisms are involved. The amount of physical activity recommended to maintain healthy weight is greater for those having lost weight than those not previously overweight [36]. The association between adipose tissue hypercellularity and leptin deficiency in obese persons who have lost weight is postulated to affect energy balance and promote the accumulation of lipid in adipocytes [37].

Among the earliest reports of progressively greater quantile dependence of adiposity to its risk factors was our paper that showed that reductions in BMI and circumferences of the waist, hip and chest per km/wk run were progressively greater for increasing percentile of their distribution [10]. Those analyses employed least squares regression to estimate the slopes of the ith percentile when the data was partitioned into deciles of running distance. This least-squares approach yielded results that are entirely consistent with quantile regression (unpublished results), except that the least-squares approach produced smoother plots of the slopes vs. the percentile of the dependent variable. Prior to that, Smith et al had reported that greater time spent before a television or computer monitor tended to raise BMI for the lower and middle percentiles of the distribution, but not in the higher percentiles [38]. Subsequently, others have employed quantile regression to describe the associations of the percentiles of BMI to its risk factors, and in many cases the effect being greater for the higher percentiles of the BMI distribution. McLaren et al reported that the inverse association between education and BMI was particularly strong among heavier women [22]. Terry et al reported that maternal weight gain was associated with their offspring's BMI for the higher percentiles (≥75^th^) of offspring's BMI distribution, but not lower percentiles of the distribution [17]. Beyerlein et al reported that maternal BMI, maternal smoking during pregnancy, weight gain during the first two years of life, television viewing time, and low parental education all showed greater effect for the higher percentiles of the offspring's BMI distribution [18]–[20]. Classen also reported that intergenerational persistence of mother-offspring BMI is strongest at higher levels of BMI [23]. Beyerlein et al reported that genetic risk for excess body weight in children is greater among fatter children [21]. Our results demonstrate the increasing influence of obesity risk factors with increasing percentiles of the BMI distribution in two separate samples and for both males and females, and formally test the significance of the progressively greater effect using linear contrasts. In addition, they show increasing influence of diet for the higher percentiles of the BMI distribution, and provide further confirmation the phenomenon for education and parental obesity.

### Limitations

The limitations of these analyses warrant acknowledgement. These data are cross-sectional, so that cause and effect cannot be proven. Our use of the terms “increase” and “decrease” are strictly in a mathematical context of the functional relationship between BMI and its risk factors. BMI is a convenient, but indirect, estimate of adiposity that may underestimate adiposity in older and younger vis-∧-vis middle-aged adults [39]. Although the percent of body fat in women is greater than in men for a given BMI, all of our analyses showed consistent results within each sex category. The assessment of dietary intake used in these analyses is limited compared to four- or seven-day dietary records and excludes potato chips, potatoes, sugar-sweetened beverages, vegetables, whole grains, and nuts that may contribute to weight gain [40]. However, this assessment of meat and fruit intake compares favorably with their assessment using four-day food records, and has been shown to yield consistently significant positive relationships between BMI and reported meat intake in 18 separate subpopulations, and significant inverse relationship between BMI and reported fruit intake in 14 out of 18 subpopulations [41], [42]. It is also acknowledged that the sample may not be exactly representative of the general population, but that the processes promoting weight gain are not expected to differ fundamentally from those of the general population. The samples are generally better educated, less sedentary, and less diverse than the general population.

Prevention is a prominent feature in the public health policies of most diseases. The current results suggest that intervening to prevent excess weight gain may not only affect the disease itself (obesity) but also its cause (the contribution of excess weight as a fundamental component of the obesogenic environment). Physical activity appears to be an effective tool in maintaining a healthy weight, and public health efforts to promote physical activity in the prevention of obesity may be best targeted at maintaining a low BMI in persons who are currently lean. Obesity is the second leading cause of preventable death [43], and increases the risks for all cause mortality, coronary heart disease, stroke, gallbladder disease, osteoarthritis, hypertension, dyslipidemia, type 2 diabetes, sleep apnea and respiratory problems, and cancers of the endometrium, breast, prostate, and colon [9]. Even within the healthy weight range, greater weight is associated with significantly increased risk for hypertension, high cholesterol, and diabetes [44], [45]. This information may be helpful in advocating weight control in the young and lean who are likely unaware of the insidious nature of weight gain. Historically, the widespread availability of inexpensive, palatable, energy-dense foods marked a major cultural achievement. The current obesity epidemic is an unexpected consequence of this accomplishment.

## Methods

### Ethics statement

The study protocol was reviewed by the University of California Berkeley committee for the protection of human subjects, and all subjects provided a signed statement of informed consent.

The analyses were restricted to the inadequately active men and women of the National Walkers' Health Study (Study 1) [11], [12] and the National Runners' Health Study (Study 2) [13], [14] whose energies expended by walking and running were less than 2.53 MET*hours/day (one MET or metabolic equivalent is approximately the energy expenditure of being at rest, or oxygen consumption of 3.5 ml O_2_•min^−1^•kg^−1^ [46]), as estimated from the compendium of physical activities published by Ainsworth et al [46]. This cut point corresponded to 75% of the energy expenditure or activity recommended by the International Association for the Study of Obesity to prevent the transition to overweight or obesity (energy equivalence of approximately 45 to 60 minutes per day of moderate activity [47]), and to an even smaller percentage of the Institute of Medicine's 2002 report (60 min of moderate- to vigorous-intensity activity on most days of the week [36]). This cut point was chosen to minimize the influence of physical activity while maximizing the sample size for the greatest statistical power, and was chosen prior to analyses. In addition, the data were statistically adjusted to remove any residual effects of physical activity (see below). The original cohorts were recruited through the distribution of a health and activity questionnaire to participants of footrace events and subscribers to *Runner's World* and *Walking Magazine* between 1991 and 2001 for the National Runners' Health Study, and between 1996 and 2001 for the National Walkers' Health Study. Although the samples were not necessarily representative of the general population, the basic physiological processes giving rise to unhealthy weight in these cohorts is not expected to fundamentally differ from those in the general population. The analyses presented were restricted to non-smoking subjects with complete data required for the analyses.

As part of their baseline survey, each participant completed a two-page mailed questionnaire that included demographics (age, race, education), exercise, height, weight, and diet. The subjects' BMIs were calculated as the weight in kilograms divided by height in meters squared. Self-reported height and weight from the questionnaire have been found previously to correlate strongly with their clinic measurements (r = 0.96 for both) [10]. Education was obtained from a simple request that the participant provide their “years of education (example HS = 12; B.S. or B.A = 16; M.S. or M.A. = 18; Ph.D. or M.D. = 20)”. The MET values provided in the compendium of physical activities [46] translate into an exercise dose that is solely a function of distance (1.02 kcal/kg or MET•hours per km). The energy expended by walking was computed by converting the reported distance into duration (i.e., distance/mph) and then calculating the product of the average hours walked per day and the MET value corresponding to their reported pace [46].

Intakes of meat and fruit were based on the questions “During an average week, how many servings of beef, lamb, or pork do you eat”, and “…pieces of fruit do you eat”. Participant provided a numerical response to the number of serving of meat or pieces of fruit consumed per week. The midpoint was used when a range of intakes was specified by the participant. Correlations between these responses and values obtained from 4-day diet records in 110 men were r = 0.46 and r = 0.38 for consumptions of meat and fruit, respectively. These values agree favorably with published correlations between food records and more extensive food frequency questionnaires for red meat (r = 0.50), and somewhat less favorably for fruit intake (r = 0.50) [48]. It is not known whether meat and fruit were directly related to BMI, or whether meat and fruit content are simply indicators of energy-dense diets that increase the risk for weight gain. Assuming the latter, standard least squares regression analyses were used to define the linear combinations of meat and fruit intake that best described the participants' BMIs when adjusted for other covariates separately in female (2.00meat-0.12fruit) and male (1.47meat-0.31fruit) participants of Study 1, and female (1.00meat-0.06fruit) and male (0.62 meat-0.15fruit) participants of Study 2 [41], [42]. The stronger and more significant contribution of meat to BMI, and the weaker inverse association with fruit intake are entirely consistent with other published results [15]. These linear combinations define the high-risk dietary index for weight gain used in the analyses.

A four point scale of parental adiposity was assessed from the question: “Would you describe your mother (father) as: 1) lean, 2) average, 3) overweight, 4) very overweight, 5) unknown”. Standard least squares regression analyses were again used to define the linear combinations of mother's and father's BMIs that best described their offspring's BMI separately in female (0.73mother + 0.79father) and male (0.82mother + 0.59father) participants of Study 1, and female (0.47mother +0.34father) and male (0.52mother +0.37father) participants of Study 2 when adjusted for other covariates. These linear combinations define the high-risk family history index for weight gain used in the analyses [49].

### Statistical analyses

Results are presented as mean±SE or slopes±SE except where noted. With the exception of the sample descriptions of Tables 1 and 2, all analyses were adjusted for age (age and age^2^), physical activity (MET hours/week), and race.

In addition, the analyses of education were also adjusted for diet and parental adiposity (Study 1 only), the analyses of diet were adjusted for education and parental adiposity (Study 1 only), and the analyses of inheritance of parental adiposity were adjusted for education and diet. Parental adiposity was not included as a covariate in Study 2 because it was only included as a survey question for one-half of the sample.

Quantile regression analysis was used to estimate the slope of the ith percentile of the adjusted BMI distribution with diet, education, and parental adiposity [24]. Specifically, this approach estimates the BMI-risk factor slope for the 5^th^ percentile of the BMI distribution, the 6^th^ percentile of the BMI distribution,…, the 95^th^ percentile of the BMI distribution, and their associated significance level. Thus the analyses yields 91 regression slopes corresponding to the 5^th^, 6^th^, …,95^th^ BMI percentile. This was followed by estimating the “slope of the regression slopes” to test whether the relationships of BMI to the obesity risk factors increased (or decreased) significantly in relation to the population percentile, i.e., when progressing from the lowest to the highest population percentile. This was done using a linear contrast of the individual regression slopes to yield the slope of the slopes, its standard error, and its significance from zero. Specifically, the contrast was specified as (−45*slope_5th_-44slope_6th_ −43slope_7th_ …+45slope_95th_). Standard errors were estimated from 1000 bootstrapped samples. All analyses were performed using Stata (version 11, StataCorp, College Station, TX). In the text that follows, the terms “increase” and “decrease” are used in the mathematical description of a function only, and do not imply actual changes in BMI over time.
